# Strategies to reduce delays in delivering mechanical thrombectomy for acute ischaemic stroke – an umbrella review

**DOI:** 10.3389/fneur.2024.1390482

**Published:** 2024-06-17

**Authors:** D. Ameen, H. M. Dewey, H. Khalil

**Affiliations:** ^1^Faculty of Medicine, Nursing and Health Sciences, School of Medicine, Monash University, Clayton, VIC, Australia; ^2^Department of Neurosciences, Eastern Health and Eastern Health Clinical School, Monash University, Box Hill, VIC, Australia; ^3^Department of Public Health, School of Psychology and Public Health, Latrobe University, Bundoora, VIC, Australia

**Keywords:** stroke, mechanical thrombectomy, reducing delays, workflow, delay to treatment

## Abstract

**Background:**

Mechanical thrombectomy is a time-sensitive treatment, with rapid initiation and reduced delays being associated with better patient outcomes. Several systematic reviews reported on various interventions to address delays. Hence, we performed an umbrella review of systematic reviews to summarise the current evidence.

**Methods:**

Medline, Embase, Cochrane Library and JBI were searched for published systematic reviews. Systematic Reviews that detailed outcomes related to time-to-thrombectomy or functional independence were included. Methodological quality was assessed using the JBI critical appraisal tool by two independent reviewers.

**Results:**

A total of 17 systematic reviews were included in the review. These were all assessed as high-quality reviews. A total of 13 reviews reported on functional outcomes, and 12 reviews reported on time-to-thrombectomy outcomes. Various interventions were identified as beneficial. The most frequently reported beneficial interventions that improved functional and time-related outcomes included: direct-to-angio-suite and using a mothership model (compared to drip-and-ship). Only a few studies investigated other strategies including other pre-hospital and teamwork strategies.

**Conclusion:**

Overall, there were various strategies that can be used to reduce delays in the delivery of mechanical thrombectomy with different effectiveness. The mothership model appears to be superior to the drip-and-ship model in reducing delays and improving functional outcomes. Additionally, the direct-to-angiosuite approach appears to be beneficial, but further research is required for broader implementation of this approach and to determine which groups of patients would benefit the most.

## Introduction

Multiple trials have shown the benefit of mechanical thrombectomy (MT) in selected acute ischemic stroke (AIS) patients (1, 2). MT is the mainstay of therapy in patients with AIS with a large vessel occlusion (LVO) and salvageable brain tissue. Depending on the study, up to 23% of patients with AIS may be eligible for this therapy (3). When MT can be delivered earlier and faster, patients have better functional outcomes (4). Hence, delivery of this therapy as expediently as possible is a major priority. There are various factors that cause delays in delivering MT, and significant efforts have been made to reduce these delays, employing a range of strategies that can be implemented in the pre-hospital, in-hospital and procedural setting (5).

MT is only provided in selected institutions with trained neuro-interventionalists known as Comprehensive Stroke Centres (CSC). The appropriate model of care for transportation of eligible patients to these centres is not well established. Significant research has been performed to determine the optimal method of identifying patients who may be potentially eligible for MT and transporting these patients to the appropriate CSC for MT, in a timely manner. Two main models have been described widely in the literature: the first being drip-and-ship and secondly the mothership model. Drip-and-ship refers to patients who are initially transported to centres without capability for MT, known as Primary Stroke Centres (PSC), for rapid diagnostic imaging and administration of IV thrombolysis (IVT), followed by transport to the nearest CSC for MT. In contrast, the mothership model refers to transport of patients directly to CSC to minimize the time to MT (6).

A vast array of tactics has been proposed and studied to reduce delays in delivering MT and published in several systematic reviews. However, to date, there is no summary that details these strategies, and their effectiveness. Hence, we performed an umbrella review of published systematic reviews to identify strategies to reduce delays in delivery of MT and their effectiveness in reducing workflow delays and improving patient outcomes. The results of this umbrella review have the potential to improve practice and identify areas of further research in this important topic.

## Methods

The protocol for this umbrella review was registered in PROSPERO (CRD42023482400). We followed the JBI methodology for Umbrella reviews and the PRISMA guidance for reporting the review (7, 8).

### Eligibility criteria

#### Inclusion criteria

This umbrella review considered only systematic reviews of primary studies. This included systematic reviews containing either experimental or observational studies. Systematic reviews were included if they explored a strategy that can be used to reduce delays in the delivery of mechanical thrombectomy. Included systematic reviews must have reported time-related outcomes (e.g., door-to-perfusion time) or function related outcomes, either qualitatively or quantitatively.

#### Exclusion criteria

Studies that were not systematic reviews were excluded. Studies published in non-English and conference abstracts were also excluded.

### Information sources/search strategy

The search was conducted on the 27th of September 2023. The databases searched included Ovid MEDLINE, Embase, Cochrane library and JBI. Studies published prior to 2000 were excluded as MT’s suitability in stroke patients began to be formally assessed at this time.

Among the four databases used for peer-reviewed articles, keywords were individually mapped to subject headings to allow searching in the different databases and also searched as keywords in general entries. Filters were applied to limit the retrieval of articles to only those that were peer-reviewed and in the English language. A manual hand-search of the literature to identify any other systematic reviews not previously identified from the search strategy was conducted. A full search strategy can be found in Supplementary material.

### Selection process

**Figure 1:**
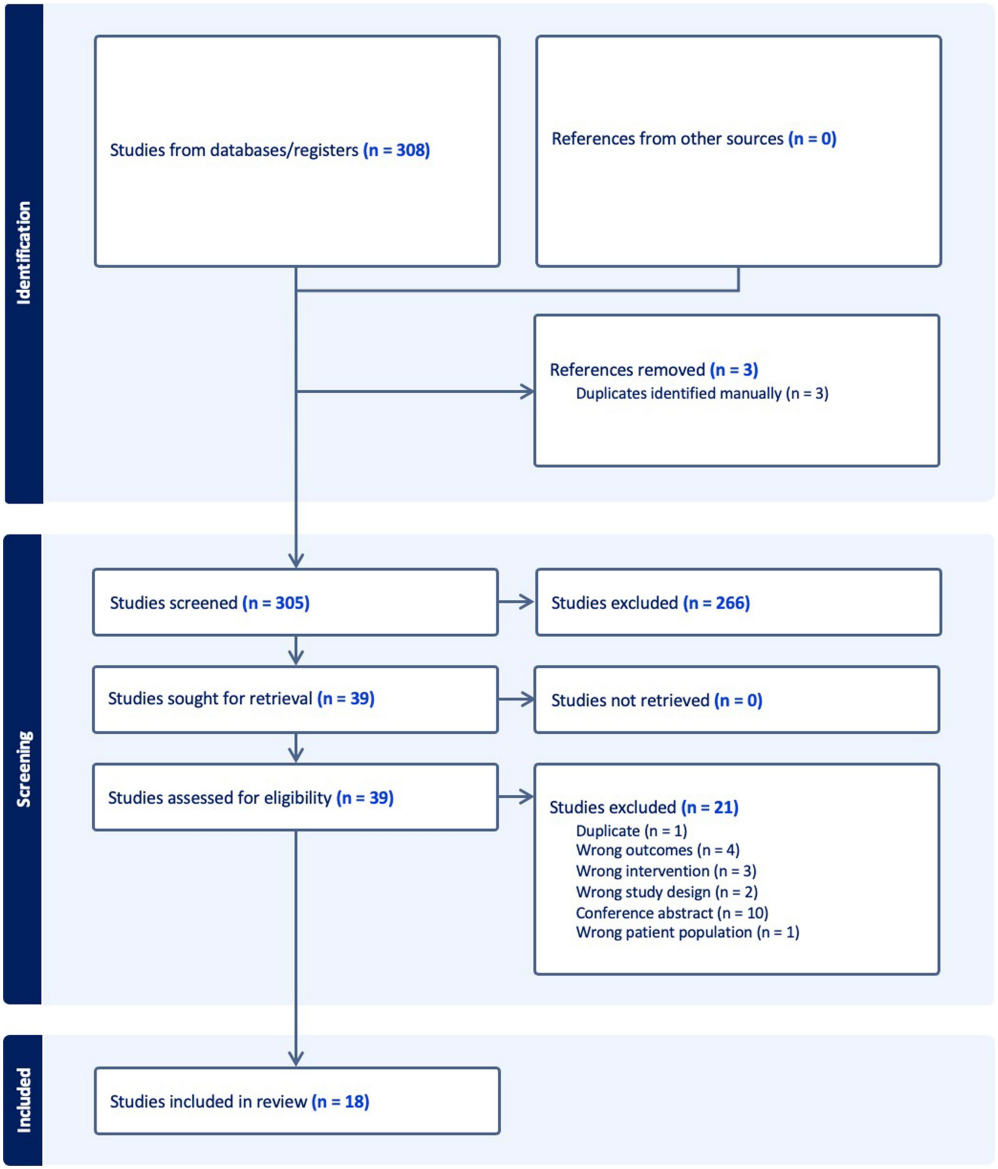
PRISMA flow chart.

Titles and abstracts were screened by 2 independent reviewers (DA, HK), and potentially eligible reviews were retrieved in full and assessed against the inclusion criteria. Full text reviews that did not meet the inclusion criteria were excluded and the reasons for exclusion are detailed in Figure 1.

### Quality and risk of bias assessment

Selected reviews were critically appraised by 2 independent reviewers (DA, HK) using the standardized JBI critical appraisal checklist for systematic reviews (8). The following quality thresholds were used: low quality (0 to 33% of criteria met), medium quality (34 to 66% of the criteria met), and high quality (67% or more of the criteria met). “Not applicable” criteria were excluded from estimates of study quality. Only medium- and high-quality reviews were included in the umbrella review.

### Data collection process and data items

Data were extracted from the reviews by using a standardized data extraction tool (Covidence) by one reviewer and extraction was checked by a second reviewer. The data extracted included specific details about the participants, setting and context, number/types of studies included, type of intervention, outcomes reported, overall effectiveness (in terms of function or time related outcomes, e.g., door-to-reperfusion time), heterogeneity in the meta-analysis if relevant and summary of findings.

Where disagreement arose between reviewers at any stage of the process, this was resolved through discussion.

### Synthesis methods

Tabular presentation of the data has been undertaken where the overall effect of each strategy from the systematic review (SR) is assessed based on the quantitative or qualitative data reported in the SR’s. For each outcome measure, a visual “stop-light” indicator was developed, where green indicates the intervention is beneficial, amber where there is no difference to the comparator and red suggests the intervention is detrimental, as per the JBI methodology of systematic reviews (8).

## Results

### Study inclusion

The search identified 308 references from the databases searched. Following removal of two duplicates leaving a total of 306 studies. Based on initial screening of the 306 studies for title and abstract, 288 studies were excluded, and 38 full texts were assessed for eligibility. A total of 21 SRs were excluded as they did not meet the inclusion criteria, leaving a total of 17 SRs for inclusion as shown in Figure 1.

### Quality and risk of bias assessment

The quality assessment results for the included 17 reviews are summarised in Supplementary Table S1. All reviews were classified as high quality, scoring between 90–100%. 16 reviews scored 100%, with the other one review scoring 90%.

### Characteristics of the included reviews

All included reviews were published after 2016, with the majority being published after 2021 as detailed in Table 1.

**Table 1:** Overview of included reviews.

Author (year)	Study design	Types of studies included in the review	Number of Studies included	Number of participants	Description of intervention type. e.g. Org	Outcome measures	Functional outcome results	Time-related outcome
Bothelo (2022)	Systematic review	Observational	128	Not reported	Pre-hospital (mobile stroke unit, point of care lab testing, telemedicine)	Qualitatively reported in time and function.	Qualitatively reported to be improved	Qualitatively reported to be improved
Brehm (2022)	Systematic review and meta-analysis	RCTs and observational studies	8	1938	In-hospital (direct to angiography suite)	Time-related: door-to-groin door-to-reperfusion Functional outcome: mRS 0–2 at 90 days	the odds of a good functional outcome were higher in the intervention group than in the control group [odds ratio (OR): 1.38, 95% CI: 0.97–1.95]	Meta-analysis showed a significant difference of median door-to-groin times of 29.0 min [95% confidence interval (CI): 14.3–43.6; *p* < 0.001] and of median door-to-reperfusion times of 32.1 min (95% CI: 15.1–49.1; *p* < 0.001) in favour of DTAS
Chowdhury (2021)	Systematic review and meta-analysis	RCTs and observational studies	26	Not reported	Pre-hospital strategies (mobile Stroke Unit, Pre-notification relevant stroke teams, telemedicine, point of care lab testing, use of pre-hospital stroke scales, pre-hospital thrombolysis) Organizational models (LVO bypass/mothership)	Time-related outcome: door-to-reperfusion	N/A	LVO bypass—(Door-to-perfusion SMD = −0.43, 95% CI = −1.32 to 0.46) not statistically significant MSU—(Door-to-perfusion SMD = −1.17, 95% CI = −1.48 to −0.86) statistically significant Improved IVT triage — (−0.30, 95% CI = -0.97 to 0.38)
Ciccone (2019)	Systematic review and meta-analysis	RCTs and Observational studies	36	4,127	Pre-hospital (organizational models - mothership vs. drip-and-ship)	Time related: onset-to-puncture time Functional outcome: mRS 0 to 2	Mothership vs. drip and ship: meta-analysis did not show a statistically significant difference in favourable functional outcome (OR 0.96; 95% CI 0.73–1.25)	Mothership vs. Drip and ship: A meta-analysis was not performed however it was evident that onset-to-puncture time was shorter in the mothership group compared to drip-and ship
Galecio-Castillo (2023)	Systematic review and meta-analysis	RCTs and Observational studies	12	2,890	In-hospital (direct to angiosuite approach)	Time related outcome: door-to-puncture time door-to-reperfusion time LKN-to-reperfusion time Functional outcome: mRS 0 to 2 at 90 days	Intervention group had greater odds of achieving favourable functional outcomes at 90 days when compared with patients following standard workflow (47.3% vs. 34.9%; OR 1.58, 95%CI 1.16 to 2.14).	Intervention group had mean reduction of 35 min door-to-puncture time compared with patients following standard workflow (MD −35.09, 95%CI −49.76 to −20.41; *p* < 0.00001) Reduction in door-to-reperfusion 31 (MD −32.88, 95%CI −50.75 to −15.01; *p* < 0.00001) LKN-to-reperfusion time (MD −58.74, 95%CI −82.76 to −34.71; *p* = 0.26)
Ismail (2019)	Systematic review and meta-analysis	RCTs and Observational studies	8	2068	Pre-hospital (organizational models - mothership vs. drip-and-ship)	Time-related: symptom onset to puncture symptom onset to reperfusion Functional: mRS 0 to 2 at 90 days	Mothership had better functional outcomes then drip-and-ship uRR = 0.87, 95% CI 0.81 to 0.93	In favour of mothership model: symptoms-onset-to-puncture (mean difference = 83.05 min, 95% CI –89.09 to 77.01) and symptoms onset to successful reperfusion (mean difference = 94.33 min, 95% CI –100.42 to 88.24)
Janssen (2019)	Systematic review and meta-analysis	RCTs and Observational studies	51	8,647	Various interventions including pre-hospital, in-hospital, teamwork, procedural, miscellaneous	Time-related: time-to-treatment Functional: mRS 0 to 2 at 90 days	The combined intervention group had a higher likelihood of favourable outcome (absolute risk difference, 12.2%; RR, 1.39; 95% CI, 1.15–1.66; *p* < 0.001) in comparison with controls.	Anaesthetic management The weighted difference in mean time to treatment was 12 min (95% CI, 6–17; *p* < 0.001) for anaesthetic management (in favour of local or conscious sedation compared to general anaesthesia) Pre-hospital management 37 min (95% CI, 22–52, *p* < 0.001) In-hospital patient transfer 41 min (95% CI, 27–54, *p* < 0.001) Teamwork 47 min (95% CI, 28–67, *p* < 0.001) Feedback 64 min (95% CI, 24–104, *p* = 0.002)
Katsanos (2023)	Systematic review and meta-analysis	Observational	6	1723	In-hospital (giving thrombolysis in PSC prior to transfer to CSC)	Time-related: onset-to-groin Functional: mRS 0 to 2 at 90 days for a “good” outcome	patients receiving IV thrombolysis at a PSC before MT transfer had a higher likelihood of 3-month good outcome (crude OR = 1.62, 95% CI 1.15–2.29; I^2 = 47%)	The mean onset-to-groin puncture time did not differ between the 2 groups (mean difference: −20 min, 95% CI −115.89 to 76.04)
Mackenzie (2021)	Systematic review and meta-analysis	RCTs and observational studies	31	49,154	Pre-hospital (organizational models - mothership vs. drip-and-ship)	Time related outcomes: onset-to-puncture time Functional outcomes: mRS 0–2 for good functional outcome mRS 0–1 for excellent functional outcome	Trend toward a higher probability of achieving mRS score 0–2 among directly admitted patients (mothership), but this was not statistically significant. However, when assessing excellent functional outcomes (mRS scores 0 to 1), direct admission was associated with significantly increased excellent functional outcomes (RR 1.20 [95% CI 1.009, 1.32], *p* < 0.001)	onset-to-puncture time was significantly longer (by 80.7 min) for transferred patients (drip-and-ship) (*p* < 0.001) compared to directly admitted patients (mothership)
Mohamadden (2022)	Systematic review and meta-analysis	RCTs and observational studies	7	1971	In-hospital – direct to angiosuite approach	Time outcome: door to puncture time (DTP) door to reperfusion Functional outcome: mRS 0–2—functional independence	Higher functional independence rate in intervention group compared with conventional approach (RR, 1.25 [95% CI, 1.02–1.53]; *p* = 0.03)	Intervention group had a reduced DTP time (mean difference, −30.76 min [95% CI, −43.70 to −17.82]; *p* < 0.001) And reduced door-to-reperfusion time: door-to-reperfusion time (mean difference, −33.24 min [95% CI, −51.82 to −14.66]; *p* < 0.001).
Ouyang (2016)	Systematic review and meta-analysis	RCTs	9	2,441	Procedural – anaesthetic management (conscious sedation vs. general anaesthesia)	Functional outcome: mRS 0–2 at 90 days	MT performed under conscious sedation yielded better functional outcome (OR, 2.08; 95% CI, 1.47–2.96; I2 = 0%)	N/A
Rangel (2022)	Systematic review and meta-analysis	Observational	14	2,277	Pre-hospital (prenotification of ED team, CT technologist, and stroke team by EMS) Teamwork (early communication between ED team and stroke team regarding plan of care + Stroke team notification system) In-hospital (single-call activation systems imaging – modified CT flows)	Time-related: door-to-puncture time	N/A	The pooled DTP improvement was SMD (1.37; 95% confidence interval, 1.20–1.93; s2 ¼1.09; *p* < 0.001). Specific interventions: Stroke tools: SMD 1.42 95% CI 1.01 to 1.83 Rapid triage protocol and stroke team notification SMD: 2.44 95% CI -0.96 to 5.83 Modified CT flow: SMD: 1.96 95% CI 1.15 to 2.77 Team-based approach SMD 0.35 (95% CI 0.08 to 0.61) Combination of interventions SMD 0.79 95% CI 0.50 to 1.08
Romoli (2020)	Systematic review and meta-analysis	RCTs and Observational	18	7,017	Pre-hospital (organizational models - mothership vs. drip-and-ship)	Functional independence: mRS 0–2 at 90 days	functional independence at 90 days, mothership paradigm was superior to drip-and-ship model (OR, 1.34; 95% CI, 1.16 to 1.55; I2 = 30%). Rates of functional independence: 53% in mothership 47% in drip-and-ship NNT of 29 in favour of mothership mode	N/A
Shaban (2022)	Systematic review and meta-analysis	Observational	6	945	Procedural (trans-radial vs. trans-femoral approach)	Time-related outcome: puncture-to-reperfusion time (median time from arteriotomy to TICI≥2b)	N/A	Meta-analysis demonstrated no significant difference in Puncture-to-reperfusion time between TRA and TFA (SMD − 0.14, 95% CI − 0.42 − 0.14; *p* = 0.323)
Shlobin (2022)	Systematic review	Observational	40	N/A	Pre-hospital (LVO triage/bypass using AI tools)	Functional outcome: qualitatively reported	Qualitative reported to be improved	N/A
Zhan (2023)	Systematic review and meta-analysis	RCTs only.	2	1,533	In-hospital (imaging protocol for patients in the extended time window)	Time-related outcome: time last seen well to puncture Functional outcome: mRS 0–2 at 90 days	There was no significant difference in mRS scores between the NCCT ± CTA and CTP groups at 90 days of follow-up (RR: 0.98; 95% CI: 0.84, 1.15)	Patients in the NCCT ± CTA group had a significantly shorter time from last seemed well to puncture than those in the CTP group (SMD: −0.14; 95% CI: −0.24, −0.04).
Zhao (2021)	Systematic review and meta-analysis	RCTs and Observational	19	4,205	Pre-hospital (organizational models - mothership vs. drip-and-ship)	Functional outcome: mRS 0–2 at 90 days	directly admitted patients had higher chances of achieving a favourable functional outcome at 3 months compared with those secondarily transferred to CSCs (OR = 1.26; 95% CI, 1.12–1.42; *p* < 0.001)	N/A

Of the 17 included studies, the total number of participants ranged from 945 (9) to 49,154 (10). 15 of the included studies comprised both a Systematic Review and a Meta-analysis, with the remaining two studies (11, 12) consisting of only a systematic review as meta-analysis was not possible due to heterogeneity of the studies. 10 SRs included both randomized controlled studies (RCTs) and observational studies (5, 10, 11, 13–20), five included only observational studies (9, 11, 12, 21, 22), and two included only RCTs (23, 24).

### Reported outcome measures

The time-related outcomes that were reported frequently included door-to-puncture time for studies assessing strategies aimed at reducing in-hospital delays (10, 15, 21). Symptom onset-to-puncture time was also used for pre-hospital strategies (16). Time-to-treatment was also used (5).

For studies that assessed functional outcome, the proportion of patients achieving a good functional outcome was assessed according to the modified Rankin Scale (mRS) at 90 days. mRS 0 to 2 was classified as a good functional outcome and mRS 0 to 1 was classified as an excellent functional outcome in all included studies that used mRS as a measure for functional independence.

For the studies where a meta-analysis was not performed, outcomes were qualitatively discussed (11, 12).

## Findings of the review

A summary of the findings of the review is detailed in Supplementary Tables S2, S3 respectively.

### Procedural

Two systematic reviews assessed procedural factors that may fasten time to thrombectomy and reperfusion (Supplementary Tables S2, S3).

As seen in Supplementary Table S3, the use of non-general anaesthesia compared to general anaesthesia reduced time to reperfusion in Janssen et al. (5) and showed an improvement in functional outcomes in Ouyang et al. (24). The use of trans-radial vs. trans-femoral arterial puncture for MT did not change time to reperfusion (9).

### Prehospital management

Seven SRs assessed the impact of strategies to reduce delays in MT on functional outcomes and five SRs addressed strategies to improve functional outcomes. The use of a mothership compared to drip-and-ship model, was found to be superior in improving functional outcomes in five (5, 10, 13, 14, 16) of six SRs that compared these models. The remaining SR by Ciccone et al. (18) showed no improvement in functional outcome using this model. Other strategies that were found to be beneficial in improving both time and functional outcomes included pre-notification of the ED team, CT technologist, and stroke team by Emergency Medical Services (EMS) (5), mobile stroke treatment unit with CT scanner (5, 12, 18), point of care laboratory testing, vascular neurologist available via telemedicine, CT cerebral angiography (CTA) at PSC vs. at CSC and air transfer as compared to ground transfer (5). AI tools for LVO triage (11) was assessed to be beneficial in improving functional outcomes (11).

### In-hospital transfer

Five SRs (5, 11, 15, 17, 20) addressed the benefits of functional outcomes for strategies to reduce delays in MT, and six SRs (5, 10, 17, 20, 21, 23) addressed strategies to reduce delays in MT in terms of time-related outcomes. The most frequently reported strategy that was assessed in four SRs was “Direct to angio-suite” (5, 15, 17, 20). All SRs that reviewed this approach with time metrics showed an improvement in time-related outcomes, and three of the four SRs (5, 15, 17) that assessed this in terms of functional outcomes showed improvements. The remaining SR by Brehm et al. (20) showed no statistically significant difference in functional outcomes for patients who went direct to angiography suite versus those who did not.

Modification of CT stroke protocols [e.g., omitting CT Perfusion imaging (CTP)] in the extended time window improved the time to MT, however, did not alter functional outcomes (23). Other strategies that were found to be beneficial in terms of reducing time to mechanical thrombectomy included: using novel stroke tools to identify LVO and using a single room for CT, angiography and MT (21).

### Teamwork

Two SRs addressed four strategies regarding teamwork to improve time to MT (5, 21). These strategies included: early communication between the ED team and stroke team regarding plan of care, early activation of the neuro-interventional team, parallel processing from ED/hospital ward to CT (patient evaluation, lab results and management) and finally, parallel processing from CT to angio-suite (where the neuro-interventional team meets patients at CT and treatment decisions are made while angio-suite is set up). These strategies were shown to reduce the time to MT by Janseen et al. (5) and Rangel et al. (21). However, no SRs reported the effectiveness of these strategies alone for improvement in functional outcomes.

### Feedback

Only one SR by Janseen et al. (5) discussed strategies in the category of “feedback.” These strategies included: providing the ED, stroke, and neuro-interventional team with feedback regarding time-outcomes, and a digital system for real-time monitoring of onset to puncture time. These showed improvements in reducing the time to MT.

### Other

Rangel et al. (21) and Jansen et al. (5) discussed strategies categorized as “other” for functional outcomes and three discussed strategies in “other” for time related outcomes. The following interventions showed an improvement in reducing the time to MT: limiting non-essential interventions, standardized angiography instrument and catheter set up for all MT devices, no groin shaving for MT, standard operating procedures for intubation at ICU prior to MT, standard operating procedures for MT, not waiting for the effect of IV tPA vs. waiting prior to MT.

Katsanos et al. (22) assessed the role of IV thrombolysis prior to transfer from PSC and showed an improvement in functional outcomes for those who received IV thrombolysis compared to those that did not, without affecting the time to MT.

## Discussion

To our knowledge, this is the first umbrella review examining the various interventions to prevent delays in MT. A range of strategies have been used to reduce delays in delivery of MT at various stages including: pre-hospital [by EMS and system-based interventions (mothership vs. drip-and-ship)], in-hospital strategies (including expedited patient assessment, imaging, and neuro-intervention delivery), teamwork strategies between staff, training and feedback for staff.

### Pre-hospital strategies

The optimal organizational model for the delivery of MT is a topic that has been extensively studied. The results of this review suggest that it may be reasonable to recommend mothership compared to drip-and-ship given that five SR’s (5, 10, 13, 14, 16) showed positive improvements in both time and function related outcomes. This was based on results of systematic reviews published between 2019 to 2021, which were more recent than the European Stroke Organisation guidelines published in 2019 (25). These guidelines do not give a clear recommendation of one approach over the other due to poor evidence quality (25).

However, whilst the mothership model demonstrated superiority in multiple SRs, travel time and resource access may prevent translation of these results, particularly in geographically spread countries with large rural populations. Hence, it may be difficult to make a strong recommendation of the mothership model in clinical practice.

The use of the mothership model may be further augmented by AI tools that aid in improving diagnoses and triage decision making, allowing for accurate identification of LVO’s in the pre-hospital setting and hence transportation of these patients to eligible centres (11).

Another important consideration when considering the drip-and-ship approach is the delivery of thrombolysis, which remains to be an important part of the management of patients presenting with an LVO to a PSC. Katsanos et al. (22) assessed the role of administering IVT in PSC prior to transfer to a CSC for MT. They found that despite the additional step in management, the time to thrombectomy in such patients did not increase, and functional outcomes were improved in this group. Hence, given the important role of thrombolysis, the strategies in this review should be used alongside other interventions that reduce delays in thrombolysis with improvements in door-to-needle times, as these are also likely to reduce delays to MT, both in PSC and CSC (26).

### In-hospital strategies

A vast variety of in-hospital interventions showed improvements in both functional and time-related outcomes.

Implementation of appropriate workflow modifications in imaging is an important intervention to reduce delays. Workflow modification produced significant reductions in time to thrombectomy. Modifications to CT workflow included a “no turn back” approach from the CT scan suite to angiography suite, and altering flow so that imaging and treatment are performed in the same room (21), all of which improved time to thrombectomy. Additionally, the role of advanced imaging techniques (CT-perfusion and CT angiography) should be considered in this paradigm. The use of these advanced imaging techniques, particularly in the extended time window (6–24 h), whilst causing delays in thrombectomy for eligible patients, does not result in deleterious functional outcomes. Hence, particularly in this group, advanced imaging modalities remains an important part of management.

Moreover, our review findings suggest the efficacy of the direct-to-angio-suite approach (17). However, it’s broader implementation remains constrained by the absence of precise pre-hospital metrics for identifying LVO strokes. Whilst the pre-hospital identification of LVO, may be achieved using the NIHSS (National institute of Health Stroke scale) with moderate accuracy (sensitivity 81.0%, specificity 76.6% when NIHSS cut off value is 7) (27), there is still likely to be a significant number of patients undergoing unnecessary angiography, due to inadequate accuracy with this approach, and possible misidentification of stroke mimics.

Hence, this review underscores the importance of further research for tools that can accurately identify LVO’s in the pre-hospital setting, prior to wide-spread adoption of this approach. Additionally, further research is required to identify which groups of patients identified in the pre-hospital field would most benefit from the direct-to-angio-suite approach.

Choice of Anaesthetic management affects time to thrombectomy and functional outcomes. Based on the findings of the review, where appropriate, using local anesthesia or conscious sedation in comparison to general anaesthesia is an important recommendation that improves time to thrombectomy and functional outcomes.

Furthermore, this review demonstrated that a time-saving effect of over 1 h (5) could be achieved by providing feedback on time intervals and outcomes to the entire stroke team regularly. This can be implemented through regular staff online bulletins at meetings where relevant members of the stroke team are attending (vascular neurologists, neuroradiologists and neurointerventionalists).

Additionally, optimization of in-hospital teamwork can be achieved by using parallel instead of sequential processing and early activation of all team members. This can be facilitated by early communication between the ED, stroke teams, and subsequently with neuro-interventionalist and neuroradiologists. Developing standard operating procedures and multidisciplinary protocols might also support this strategy. Such interventions can be employed at any institution centre, and according to Janseen et al., may result in a time saving effect of 47 min (5).

Moreover, this review highlighted the importance of assessing both improvement in time-related outcomes and functional outcomes when assessing strategies to reduce delays to thrombectomy. In the included reviews, these were not always consistent, and reduction in time did not always result in improvement in functional outcomes for patients.

This review has several limitations. The umbrella review methodology, while effective in synthesizing diverse studies, poses limitations by sacrificing data granularity. Consequently, drawing conclusions from the synthesized evidence becomes challenging. Additionally, there was significant heterogeneity in the reporting of outcome measures which meant that performing a meta-analysis was not possible. Moreover, while this umbrella review mapped the effectiveness of interventions, it was not possible to make head-to-head comparisons between the different interventions to identify the most effect strategies for reducing delays to MT. Finally, a common limitation in searching for studies is that relevant studies may have been missed or omitted through the search and screening process, which may have impacted our results.

This review has highlighted multiple areas of direction for future research. There was limited research conducted in the areas of: choice of anaesthetic in different populations, teamwork, feedback to staff and other interventions such as the presence of standard operating procedures and role of IV thrombolysis at PSC prior to transfer. Additionally, the heterogeneity of outcomes presented in the reviews precluded secondary analysis of the data. Hence, ensuring standard reporting of outcome measures (e.g., symptom onset to puncture, door-to-puncture) will enable future researchers to compare the effectiveness of different workflow improvements. No included reviews assessed the differences in neurointerventional training in accepting cases, be it amongst endovascular neurosurgeons, interventional neurologists and neurointerventional radiologists, and potential delays this may introduce. The role of AI in the diagnosis of LVO stroke in the pre-hospital and in-hospital setting has not been evaluated in terms of the direct benefit on patient outcomes, and this is an active area of future research.

## Conclusion

Overall, we have highlighted strategies that can be used to reduce delays in the delivery of mechanical thrombectomy for acute ischemic stroke in the pre-hospital and in-hospital setting. The choice of the interventions will depend on several factors such as resource availability, the location of the health service and patient factors. The mothership model appears to be superior to the drip-and-ship model in reducing time delays and improving functional outcomes. Additionally, the direct-to-angio-suite approach appears to be beneficial, but further research is required to validate this process and to identify which groups of patients would benefit most from this approach.

## Data availability statement

The original contributions presented in the study are included in the article/Supplementary material, further inquiries can be directed to the corresponding author.

## Author contributions

DA: Writing – original draft, Writing – review & editing. HD: Writing – review & editing. HK: Writing – original draft, Writing – review & editing.
