# Supplementary material for: Strategies to reduce delays in delivering mechanical thrombectomy for acute ischaemic stroke – an umbrella review
Source: Front Neurol. 2024 Jun 17;15:1390482. doi: 10.3389/fneur.2024.1390482 (PMC11215205; doi:10.3389/fneur.2024.1390482)
Supplement: Supplementary file 1 [file Data_Sheet_1.docx]

**Appendix 1**

**Search strategy is shown below:**

**Databases:**JBI EBP database

Cochrane Databases of systematic reviews

Embase Classic + Embase

Ovid MEDLINE

| **#** | **Query** | **Results from 27 Sep 2023** |
| --- | --- | --- |
| 1 | Endovascular Procedures/ | 58,415 |
| 2 | Thrombectomy/ or mechanical thrombectomy.mp. | 44,901 |
| 3 | Thrombotic Stroke/ or Stroke/ or Embolic Stroke/ or Ischemic Stroke/ | 444,484 |
| 4 | brain ischemia.mp. or Brain Ischemia/ | 228,016 |
| 5 | cerebrovascular accident.mp. | 301,109 |
| 6 | Stroke/su | 6,569 |
| 7 | health services accessibility.mp. or Health Services Accessibility/ | 161,275 |
| 8 | time-to-treatment.mp. or Time-to-Treatment/ | 80,598 |
| 9 | Hospitals/ or Workflow/ or Time-to-Treatment/ or hospital workflow.mp. | 508,835 |
| 10 | Hospitals/ or Workflow/ or Time-to-Treatment/ or hospital workflow.mp. or Emergency Service, Hospital/ | 602,646 |
| 11 | patient transfer.mp. or Patient Transfer/ | 45,373 |
| 12 | door to groin.mp. | 502 |
| 13 | Endovascular thrombectomy.mp. | 5,001 |
| 14 | intra-arterial therapy.mp. | 1,297 |
| 15 | streamlining.mp. | 9,138 |
| 16 | strategies.mp. | 1,982,858 |
| 17 | 1 or 2 or 6 or 13 or 14 | 105,547 |
| 18 | large vessel occlusion.mp. | 10,710 |
| 19 | 3 or 4 or 5 or 18 | 727,592 |
| 20 | triage.mp. or Triage/ | 71,322 |
| 21 | Delayed Diagnosis/ or Diagnosis/ or diagnosis.mp. | 10,918,000 |
| 22 | systematic review.mp. or "Systematic Review"/ | 858,366 |
| 23 | 7 or 8 or 9 or 10 or 11 or 12 or 15 or 16 or 20 or 21 | 13,332,258 |
| 24 | 17 and 19 and 22 and 23 | 350 |
| 25 | remove duplicates from 24 | 308 |

Endovascular Procedures/
Thrombectomy/ or mechanical thrombectomy.mp.
Thrombotic Stroke/ or Stroke/ or Embolic Stroke/ or Ischemic Stroke/
brain ischemia.mp. or Brain Ischemia/
cerebrovascular accident.mp.
Stroke/su
health services accessibility.mp. or Health Services Accessibility/
time-to-treatment.mp. or Time-to-Treatment/
Hospitals/ or Workflow/ or Time-to-Treatment/ or hospital workflow.mp.
Hospitals/ or Workflow/ or Time-to-Treatment/ or hospital workflow.mp. or Emergency Service, Hospital/
patient transfer.mp. or Patient Transfer/
door to groin.mp.
Endovascular thrombectomy.mp.
intra-arterial therapy.mp.
streamlining.mp.
strategies.mp.
1 or 2 or 6 or 13 or 14
large vessel occlusion.mp.
3 or 4 or 5 or 18
triage.mp. or Triage/
Delayed Diagnosis/ or Diagnosis/ or diagnosis.mp.
systematic review.mp. or "Systematic Review"/
7 or 8 or 9 or 10 or 11 or 12 or 15 or 16 or 20 or 21
17 and 19 and 22 and 23
remove duplicates from 24

#### **Table S1: Critical Appraisal of Eligible Systematic Review and Research Syntheses**

| **eCitation** | **Q1** | **Q2** | **Q3** | **Q4** | **Q5** | **Q6** | **Q7** | **Q8** | **Q9** | **Q10** | **Q11** | **%**  **(Quality)** |
| --- | --- | --- | --- | --- | --- | --- | --- | --- | --- | --- | --- | --- |
| Botelho A, Rios J, Fidalgo AP, Ferreira E, Nzwalo H. 2022. | Y | Y | Y | Y | Y | U | Y | Y | U | Y | Y | 100  High |
| Brehm A, Tsogkas I, Ospel JM, Appenzeller-Herzog C, Aoki J, Kimura K, et al. 2022. | Y | Y | Y | Y | Y | Y | Y | Y | U | U | Y | 100  High |
| Chowdhury SZ, Baskar PS, Bhaskar S. 2021. | Y | Y | Y | Y | Y | Y | Y | Y | Y | U | Y | 100  High |
| Ciccone A, Berge E, Fischer U. 2019. | Y | Y | Y | Y | Y | Y | Y | Y | U | Y | Y | 100  High |
| Galecio-Castillo M, Vivanco-Suarez J, Zevallos CB, Dajles A, Weng J, Farooqui M, et al. 2023. | Y | Y | Y | Y | Y | U | Y | Y | Y | Y | Y | 100  High |
| Ghozy S, Hasanzadeh A, Kobeissi H, Abdelghaffar M, Shafie M, Beizavi Z, et al. 2023. | Y | Y | Y | Y | Y | Y | Y | Y | Y | Y | Y | 100  High |
| Ismail M, Armoiry X, Tau N, Zhu F, Sadeh-Gonik U, Piotin M, et al. 2019. | Y | Y | Y | Y | Y | U | Y | Y | N | Y | Y | 90  High |
| Janssen PM, Venema E, D. Ippel D.W.J. 2019. | Y | Y | Y | Y | Y | U | Y | Y | Y | Y | Y | 100  High |
| Katsanos AH, Sarraj A, Froehler M, Purrucker J, Goyal N, Regenhardt RW, et al. 2023. | Y | Y | Y | Y | Y | Y | U | Y | Y | Y | U | 100  High |
| MacKenzie IER, Arusoo T, Sigounas D. 2021. | Y | Y | Y | Y | U | Y | Y | Y | U | Y | Y | 100  High |
| Mohammaden MH, Doheim MF, Elfil M, Al-Bayati AR, Pinheiro A, Nguyen TN, et al. 2022. | Y | Y | Y | Y | Y | Y | Y | Y | U | Y | Y | 100  High |
| Ouyang F, Chen Y, Zhao Y, Dang G, Liang J, Zeng J. 2016. | Y | Y | Y | Y | Y | Y | Y | Y | Y | Y | Y | 100  High |
| Rangel I, Palmisciano P, Vanderhye VK, El Ahmadieh TY, Wahood W, Demaerschalk BM, et al. 2022. | Y | Y | Y | Y | Y | Y | Y | Y | U | Y | Y | 100  High |
| Romoli M, Paciaroni M, Tsivgoulis G, Agostoni EC, Vidale S. 2020. | Y | Y | Y | Y | Y | Y | Y | Y | Y | Y | Y | 100  High |
| Shaban S, Rastogi A, Phuyal S, Huasen B, Haridas A, Zelenak K, et al. 2022. | Y | Y | Y | Y | Y | U | U | Y | Y | Y | Y | 100  High |
| Shlobin NA, Baig AA, Waqas M, Patel TR, Dossani RH, Wilson M, et al. 2022. | Y | Y | Y | Y | Y | U | U | Y | U | Y | Y | 100  High |
| Zhan Z, Gu F, Ji Y, Zhang Y, Ge Y, Wang Z. 2023. | Y | Y | Y | Y | Y | Y | Y | Y | U | Y | Y | 100  High |
| Zhao W, Ma P, Chen J, Yue X. 2021. | Y | Y | Y | Y | Y | Y | Y | Y | Y | Y | Y | 100  High |
| % | 100.0 | 100.0 | 100.0 | 100.0 | 94.44 | 66.66 | 83.33 | 100.0 | 47.0 | 88.88 | 94.44 |  |

**Table S2: Strategies to reduce delays in delivering mechanical MT in terms of functional outcomes.**

| **Study name** | **Procedural components** | | **Prehospital management** | | | | | | | **In hospital management** | | | | **Teamwork** | | | | **Feedback** | | **Other** | | | | | | | | | | | | | |  |
| --- | --- | --- | --- | --- | --- | --- | --- | --- | --- | --- | --- | --- | --- | --- | --- | --- | --- | --- | --- | --- | --- | --- | --- | --- | --- | --- | --- | --- | --- | --- | --- | --- | --- | --- |
|  | non general anaesthesia | Trans-radial access (as compared to transfemoral) | Prenotification ED team, CT technologist, and stroke team by EMS | Mobile stroke treatment unit with CT scanner, point of care  laboratory testing, vascular neurologist available via telemedicine | Mothership model (as compared to drip-and-ship) | CTA at PSC (compared to at CSC) | AI tools for LVA triage | Use of telemedicine assessment by a stroke neurologist at PSC | Air transfer (compared to ground transfer) | Direct to angiography suite strategy | Using novel stroke tools to identify LVO (including AI) | Modified CT stroke protocol | Single room used for CT, angiography, and MT | Early communication between ED team and stroke team about plan  of care | Early activation neurointerventional team | Parallel processing for patient evaluation, lab testing and managing | Parallel processing from CT to angio-suite; neurointerventional team meets patient at CT, and treatment decisions are made | Education and feedback all teams | digital system for real-time monitoring of onset to puncture | | Limiting nonessential interventions (eg, ECG, chest X-ray, additional  venous access, bladder catheter placement) | | Standard angiography set for all of the devices needed for MT | | No groin shaving | | Standard operating procedure for intubation at the ICU  before MT | | Standard operating procedure for MT | | Not waiting for effect IV tissue-type plasminogen activator for 1 h | | IV thrombolysis at PSC prior to transfer | |
| Bothelo 2022 | Not reported | | | | | | | | | | | | | | | | | | | | | | | | | | | | | | | | |  |
| Brehm 2022 |  | | | | | | | | |  |  | | | | | | | | | | | | | | | | | | | | | | |  |
| Chowdhury 2021 | Not reported | | | | | | | | | | | | | | | | | | | | | | | | | | | | | | | | |  |
| Ciccone 2019 |  |  |  |  |  |  |  |  |  |  |  |  |  |  |  |  |  |  |  |  | |  | |  | |  | |  | |  | |  | |  |
| Galecio-Castillo 2023 |  |  |  |  |  |  |  |  |  |  |  |  |  |  |  |  |  |  |  |  | |  | |  | |  | |  | |  | |  | |  |
| Ismail 2019 |  |  |  |  |  |  |  |  |  |  |  |  |  |  |  |  |  |  |  |  | |  | |  | |  | |  | |  | |  | |  |
| Janssen 2019 |  |  |  |  |  |  |  |  |  |  |  |  |  |  |  |  |  |  |  |  | |  | |  | |  | |  | |  | |  | |  |
| Katsanos 2023 |  |  |  |  |  |  |  |  |  |  |  |  |  |  |  |  |  |  |  |  | |  | |  | |  | |  | |  | |  | |  |
| Mackenzie 2021 |  |  |  |  |  |  |  |  |  |  |  |  |  |  |  |  |  |  |  |  | |  | |  | |  | |  | |  | |  | |  |
| Mohamadden 2022 |  |  |  |  |  |  |  |  |  |  |  |  |  |  |  |  |  |  |  |  | |  | |  | |  | |  | |  | |  | |  |
| Ouyang 2016 |  |  |  |  |  |  |  |  |  |  |  |  |  |  |  |  |  |  |  |  | |  | |  | |  | |  | |  | |  | |  |
| Rangel 2022 | Not reported | | | | | | | | | | | | | | | | | | | | | | | | | | | | | | | | |  |
| Romoli 2020 |  |  |  |  |  |  |  |  |  |  |  |  |  |  |  |  |  |  |  |  | |  | |  | |  | |  | |  | |  | |  |
| Shaban 2022 | Not reported | | | | | | | | | | | | | | | | | | | | | | | | | | | | | | | | |  |
| Shlobin 2022 |  |  |  |  |  |  |  |  |  |  |  |  |  |  |  |  |  |  |  |  | |  | |  | |  | |  | |  | |  | |  |
| Zhan 2023 |  |  |  |  |  |  |  |  |  |  |  |  |  |  |  |  |  |  |  |  | |  | |  | |  | |  | |  | |  | |  |
| Zhao 2021 |  |  |  |  |  |  |  |  |  |  |  |  |  |  |  |  |  |  |  |  | |  | |  | |  | |  | |  | |  | |  |

**Table S3: Strategies to reduce delays in delivering mechanical MT in terms of reducing delays (time-related outcomes)**

| Study name | Procedural components | | Prehospital management | | | | | | | In hospital management | | | | Teamwork | | | | Feedback | | Other | | | | | | |
| --- | --- | --- | --- | --- | --- | --- | --- | --- | --- | --- | --- | --- | --- | --- | --- | --- | --- | --- | --- | --- | --- | --- | --- | --- | --- | --- |
|  | non general anaesthesia (compared to general anaesthesia) | Trans-radial access (as compared to transfemoral) | Prenotification ED team, CT technologist, and stroke team by EMS | Mobile stroke treatment unit with CT scanner, point of care  laboratory testing, vascular neurologist available via telemedicine | Mothership (as compared to drip-and-ship) | CTA at PSC (compared to at CSC) | AI tools for LVO triage | Use of telemedicine assessment by a stroke neurologist at PSC | Air transfer (as compared to ground transfer) | Direct to angiography suite strategy | Modified CT Stroke protocol | Using novel stroke tools to identify LVO (including AI) | Single room used for CT, angiography, and MT | Early communication between ED team and stroke team about plan  of care | Early activation neurointerventional team | Parallel processing for patient evaluation, lab testing and managing | Parallel processing from CT to angio-suite: neurointerventional  team meets patient at CT, and treatment decisions are made | Education and feedback all teams | digital system for real-time monitoring of onset to puncture time | Limiting nonessential interventions (eg, ECG, chest X-ray, additional  venous access, bladder catheter placement) | Standard angiography set for all of the devices needed for MT | No groin shaving | Standard operating procedure for intubation at the ICU before MT | Standard operating procedure for MT | Not waiting for effect IV tissue-type plasminogen activator  for 1 h | IV thrombolysis at PSC prior to transfer |
| Bothelo 2022 |  |  |  |  |  |  |  |  |  |  |  |  |  |  |  |  |  |  |  |  |  |  |  |  |  |  |
| Brehm 2022 |  |  |  |  |  |  |  |  |  |  |  |  |  |  |  |  |  |  |  |  |  |  |  |  |  |  |
| Chowdhury 2021 |  |  |  |  |  |  |  |  |  |  |  |  |  |  |  |  |  |  |  |  |  |  |  |  |  |  |
| Ciccone 2019 | Not reported | | | | | | | | | | | | | | | | | | | | | | | | | |
| Galecio-Castillo 2023 |  |  |  |  |  |  |  |  |  |  |  |  |  |  |  |  |  |  |  |  |  |  |  |  |  |  |
| Ismail 2019 |  |  |  |  |  |  |  |  |  |  |  |  |  |  |  |  |  |  |  |  |  |  |  |  |  |  |
| Janssen 2019 |  |  |  |  |  |  |  |  |  |  |  |  |  |  |  |  |  |  |  |  |  |  |  |  |  |  |
| Katsanos 2023 |  |  |  |  |  |  |  |  |  |  |  |  |  |  |  |  |  |  |  |  |  |  |  |  |  |  |
| Mackenzie 2021 |  |  |  |  |  |  |  |  |  |  |  |  |  |  |  |  |  |  |  |  |  |  |  |  |  |  |
| Mohamadden 2022 |  |  |  |  |  |  |  |  |  |  |  |  |  |  |  |  |  |  |  |  |  |  |  |  |  |  |
| Ouyang 2016 | Not reported | | | | | | | | | | | | | | | | | | | | | | | | | |
| Rangel 2022 |  |  |  |  |  |  |  |  |  |  |  |  |  |  |  |  |  |  |  |  |  |  |  |  |  |  |
| Romoli 2020 | Not reported | | | | | | | | | | | | | | | | | | | | | | | | | |
| Shaban 2022 |  |  |  |  |  |  |  |  |  |  |  |  |  |  |  |  |  |  |  |  |  |  |  |  |  |  |
| Shlobin 2022 | Not reported | | | | | | | | | | | | | | | | | | | | | | | | | |
| Zhan 2023 |  |  |  |  |  |  |  |  |  |  |  |  |  |  |  |  |  |  |  |  |  |  |  |  |  |  |
| Zhao 2021 | Not reported | | | | | | | | | | | | | | | | | | | | | | | | |  |
